# Portable eye-tracking in neurology: current uses and future perspectives in cognition

**DOI:** 10.1055/s-0046-1817035

**Published:** 2026-03-19

**Authors:** Diogo Haddad-Santos, Carolina B. Moura, Maria T. Martinez, Ana Morgado, Dagoberto Callegaro, Alex Kiderman, Renato Anghinah

**Affiliations:** 1 Universidade de São Paulo, São Paulo SP, Brazil.; 2 Irmandade Santa Casa da Misericórdia de São Paulo, São Paulo SP, Brazil.; 3 Univerisdade Federal Fluminense, Hospital Universitário Antonio Pedro, Niterói RJ, Brazil.; 4 Spryson America Inc, Pittsburgh PA, United States.

**Keywords:** Eye-Tracking Technology, Cognitive Dysfunction, Nervous System Diseases, Wearable Electronic Devices, Systematic Review

## Abstract

**Background:**

Eye tracking technology has emerged as a pivotal tool in neurology, providing objective insights into ocular motor function and cognitive processes across various neurological conditions, including mild traumatic brain injury, autism spectrum disorder, schizophrenia, attention deficit hyperactivity disorder, and neurodegenerative diseases like Alzheimer's and Parkinson's disease.

**Objective:**

The present systematic review evaluates the current applications and reliability of portable eye-tracking devices in clinical practice, highlighting their transformative potential for diagnosing and monitoring cognitive disorders.

**Methods:**

A systematic review was performed in accordance with the Preferred Reporting Items for Systematic Reviews and Meta-Analyses (PRISMA) guidelines. Observational studies using portable eye-tracking devices were included. Databases searched included PubMed, Embase, and Cochrane, with studies screened and reviewed by two independent authors. Outcomes assessed were eye movements and visual responses in neurological patients. The Critical Appraisal Skills Program (CASP) checklist was used to assess study quality and bias.

**Results:**

A total of 62 studies were identified, with 27 included after screening. The findings reveal significant advancements in device accessibility, sampling rates, and accuracy, which enhance the ability to detect subtle cognitive changes through eye movement patterns. Portable devices such as Neurolign DX 100 (Neurolign USA, LLC) and Tobii (Tobii), which is a portable video-oculography (VOG) devices including Neurolign DX 100 and Tobii systems, were highlighted for their precision and applicability in clinical settings.

**Conclusion:**

Portable eye-tracking devices show promise for detecting cognitive impairments in neurological conditions like multiple sclerosis. Their portability and ease of use facilitate widespread clinical application, making cognitive assessments more accessible and effective in early diagnosis and monitoring of disease progression.

## INTRODUCTION

Eye tracking technology has emerged as a tool in the field of neurology, offering objective insights into ocular motor function and cognitive processes. Recent studies in cognitive field include mild traumatic brain injury (mTBI), 1 autism spectrum disorder (ASD), 2 schizophrenia, 3 attention deficit hyperactivity disorder (ADHD), 4 and neurodegenerative diseases such as Alzheimer's 5 and Parkinson's disease (PD). 6 Interest in ocular motion dates back to antiquity. More than 2000 years ago, Aristotle had already described binocular coordination, noting that both eyes cannot move in opposite directions, an early recognition of the neural coupling that underlies conjugate eye movements. Centuries later, this ancient observation evolved into experimental studies linking eye movements with higher cognitive processes. In 1967, Yarbus demonstrated that gaze patterns are not random but reflect cognitive intent and mental state. 7

Since that time, the study of eye movements has advanced our understanding of movement patterns which reflect underlying cognitive processes. 8 This was proven years later through modern eye-tracking systems which now use infrared cameras and algorithms to measure gaze in real time, operating through cameras and algorithms that follow the position of the eyes. 8 9 Variations in ocular patterns, such as reduced eye fixations on relevant stimuli, can signal early changes in cognitive function, making eye tracking a valuable tool for diagnosis and intervention. 5 10 11 In addition to fixations, eye-tracking technology can detect a range of oculomotor behaviors, including saccades (rapid eye movements between points of interest), smooth pursuit (the ability to follow moving targets), vergence (eye movements that adjust for depth perception), and pupillary responses linked to cognitive load. These parameters provide a multidimensional view of neural processing, making eye tracking an increasingly important biomarker in neurology, especially in cognitive domains. 10

Historically, the technology required expensive and robust equipment, limiting its use to research settings 12 . However, devices have significantly evolved over the past few decades, transitioning from complex systems to portable and accessible solutions, including small tablets and video-oculography (VOG). 13 Recently, portable eye-tracking devices enable easy access protocols and facilitate data collection across diverse populations. 13 14 Quantitatively, portable eye-tracking devices differ from traditional ones in several key metrics. For instance, while traditional systems may operate at sampling rates of around 60 Hz, many modern portable devices can achieve rates of up to 300 Hz or more, allowing for more precise tracking of rapid eye movements. 10 This increased sampling rate and accuracy enable more detailed analysis of ocular behavior and cognitive assessment.

We aim to describe how portable eye-tracking technologies are currently used in clinical practice and to demonstrate their reliability and transformative potential in the evaluation of neurological disorders, particularly those involving cognition. We propose that incorporating portable eye-tracking into the cognitive research groups could popularize its use, raise awareness about a potential biomarker for cognitive disorders, and stimulate further research with this emerging tool. Given the growing demand for accessible and non-invasive diagnostic methods in cognitive neurology, this topic is of high relevance to clinical and research communities.

## METHODS

The present systematic review was performed and reported following the Cochrane Collaboration Handbook for Systematic Review of Interventions and the Preferred Reporting Items for Systematic Reviews and Meta-analysis (PRISMA) Statement guidelines. The protocol was prospectively registered on PROSPERO on September 20th, 2024 (CDR:420245598727).

### Eligibility criteria

Inclusion in review was restricted to studies that met all the following eligibility criteria:

- observational studies;
- use of portable device of eye-tracking;
- English full-text available studies.

The included studies should have reported at least one of the primary outcomes of interest:

- eye movements;
- visual responses;
- cognitive outcomes or domains related to eye-tracking measures, such as attention, memory, executive function, or information processing.

### Search strategy and data extraction

We systematically searched PubMed and Cochrane from inception to June 2024 with the following search strategy: ( *portable eye-tracking* OR *portable eye tracking* OR *portable video-oculography* ). The references from all included studies, previous systematic reviews, and meta-analyses were also searched manually for any additional studies. Two authors (ALM and CB) independently performed the literature search after predefined search criteria, and eventual conflicts were resolved by a third author (DHS).

### Quality assessment

We used the Critical Appraisal Skills Program (CASP) checklist to assess the methodological quality of the studies included. This tool evaluates the risk of bias across several domains, including study validity, results, and applicability. Two authors independently assess the risk of bias (ALM and CB). Two independent reviewers screened all titles and abstracts. Inter-rater reliability was assessed using Cohen's kappa, which showed substantial agreement (κ = 0.77). Discrepancies were resolved through discussion and consensus. Disagreements were resolved through a consensus after discussion with a third author.

## RESULTS

### Study selection and characteristics

**Figure 1:**
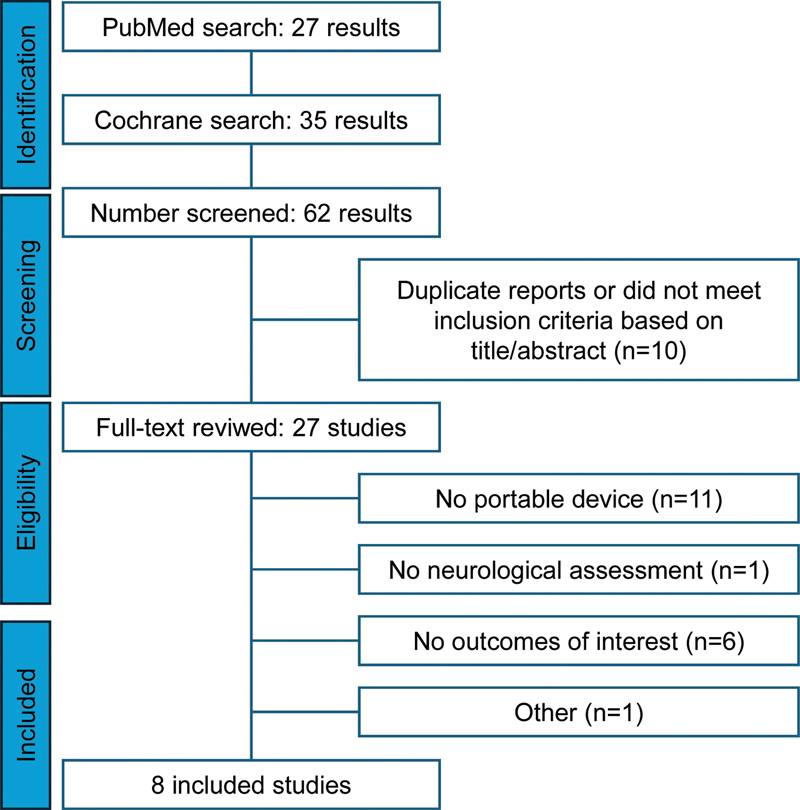
Preferred Reporting Items for Systematic Reviews and Meta-Analyses (PRISMA) flow diagram.

The initial search yielded 27 results from PubMed, and 35 results from Cochrane, with 62 results in total. After the removal of 10 duplicates, 52 articles underwent initial screening, 25 of which were excluded based on the information provided in the abstract and title, leaving 27 articles for full-text assessment for eligibility. The original studies necessarily contained information about the devices and the tests. The PRISMA flow diagram is shown in Figure 1 , detailing the reasons for the exclusion of the articles that underwent full-text assessment.

### Characteristics of the included studies and patients

The studies included in this review were published between 2016 and 2022 and comparatively investigated the use of portable eye-tracking devices in neurological conditions. The patients in these studies were predominantly affected by cognitive issues. The evaluated tasks included saccades, antisaccades, prosaccades, smooth pursuit, Oculomotor, Vestibular, Reaction Time, and Cognitive (OVRT-C) tests. Other characteristics of the included studies are shown in Table 1 .

**Table 1:** Population, devices, and tasks employed across studies

Study	Type of study	Population	Number of patients	Device	Screen	Tasks
Estrin et al., 2 2024	Observational	Autism	115	Tobii X2–60	MacBook Pro computer	Antisaccades, prosaccades
Hong et al., 3 2005	Observational	Schizophrenia	18	Applied Science Laboratories 210	Laptop computer	Saccades, smooth pursuit
Yoo et al., 4 2024	Observational	ADHD	250	SeeSo SDK	Samsung Galaxy Tab 7+	Antisaccades, prosaccades
Kullmann, 2021	Observational	General population, 18–45 years	446	Neurolign Dx 100	VOG goggles	Saccadic movements, smooth pursuit, and optokinetic responses
Fischer, 2016	Protocol	Concussion	–	Raspberry Pi 2	5-inch LCD screen	–
Newan-Toker, 2013	Observational	Possible stroke in acute vertigo and dizziness	10	ICS Impulse, GN Otometrics,	VOG	Horizontal head impulse test (h-HIT), direction changing nystagmus and test-of-skew (HINTS)
Kelly et al., 15 1990	Observational	Patients diagnosed with COVID-19 and persistent cognitive impairment after 4 weeks.	77	Neurolign Dx 100	VOG goggles	OVRT-C tests (the gaze horizontal, spontaneous nystagmus, subjective visual vertical, and subjective visual horizontal tests to assess vestibular function)
Mishra, 2020	Observational	Healthy volunteers	14	Wireless portable electrode - fabricated with aerosol jet printing (AJP)	Smartphone (Samsung Gear VR)	Ocular vergence

Abbreviations: COVID-19, coronavirus disease 2019; HINTS, Head Impulse, Nystagmus, and Test of Skew; OVRT-C, Oculomotor, Vestibular, Reaction Time, and Cognitive; VOG, video-oculography.

### Characteristics of eye-tracking devices

A total of eight studies employing eye-tracking or VOG technologies were identified, each targeting distinct populations and employing various tasks and devices. Estrin et al. (2022) 2 evaluated 115 individuals with autism using the Tobii X2–60 (Tobii) device connected to a MacBook Pro (Apple, Inc.), administering antisaccade and prosaccade tasks. Hong et al. (2005) 3 studied 18 patients with schizophrenia using the Eye-Trac 210 Eye Tracker System (Applied Science Laboratories) and a laptop computer, focusing on saccadic and smooth pursuit eye movements. Yoo et al. (2024) 4 conducted a large-scale observational study with 250 individuals diagnosed with ADHD, utilizing the SeeSo SDK (VisualCamp Co., Ltd.) on a Samsung Galaxy Tab 7+ (Samsung Electronics Co., Ltd.) to assess both anti- and prosaccades. Kullmann et al. (2021) 13 examined a general population sample of 446 adults aged 18 to 45 years using Neurolign Dx 100 (Neurolign USA, LLC) VOG goggles. The protocol included saccadic movements, smooth pursuit, and optokinetic responses. Fischer et al. (2016) proposed a protocol study for concussion assessment using a Raspberry Pi 2 (Sony Group Corporation) with a 5-inch LCD screen, although no participants or specific tasks were reported. Newman-Toker et al. (2013) assessed 10 patients presenting with acute vertigo and suspected stroke using ICS Impulse (Natus Sensory) VOG equipment. The study applied the Head Impulse, Nystagmus, and Test of Skew (HINTS) battery. Kelly et al. 15 (1990) investigated 77 post-coronavirus disease 2019 (COVID-19) patients with persistent cognitive complaints using the Neurolign Dx 100, performing OVRT-C tests including horizontal gaze, spontaneous nystagmus, and subjective visual vertical and horizontal components. Finally, Mishra et al. (2020) studied 14 healthy volunteers with a custom wireless portable electrode system fabricated via aerosol jet printing, paired with a Samsung Gear VR (Samsung Electronics Co.,) to assess ocular vergence. Across studies, device types and paradigms varied significantly, reflecting heterogeneity in hardware, screen format, and oculomotor paradigms tailored to the underlying clinical hypothesis.

### Cognitive domains assessed using portable eye tracking

#### Attention

Several studies used saccadic tasks, including pro- and antisaccades, as well as saccade prediction tests, to assess attentional control. We employed antisaccade paradigms to evaluate attention in patients with ADHD. 16 17 18 The latency and accuracy of saccadic responses served as indirect markers of attentional engagement and control. 19

#### Executive function

Antisaccade tasks were also applied in studies evaluating executive control, specifically inhibitory function. These tasks require participants to suppress a reflexive saccade toward a visual target and instead look in the opposite direction. Delayed response or failure to inhibit the reflexive saccade was interpreted as impaired executive control, relevant in conditions such as PD and schizophrenia. 3

#### Working memory

Saccade prediction tasks were used to evaluate working memory capacity. These tasks require the participant to anticipate a stimulus based on a sequence or learned pattern. Performance on these tasks reflects the ability to retain and manipulate information, a key component of working memory. 20

#### Visual processing

Smooth pursuit tasks were used in studies examining disorders like schizophrenia and multiple sclerosis (MS). 3 21 Inability to smoothly track a moving object indicated impairments in visual-motor integration and sensory prediction, functions closely tied to cognitive processing. 9

#### Inhibitory control

Several studies focused specifically on inhibitory control using anti-saccade paradigms. In patients with cognitive disorders such as schizophrenia or ADHD, increased error rates and prolonged latencies in these tasks were consistently reported. 13 15

### Risk of bias of included studies

The eight included observational studies were appraised using the CASP checklist. All studies clearly defined their research questions and used appropriate study designs. However, recruitment strategies were unclear in 2 of the studies (Hong et al., 3 2005; Mishra, 2020), and confounding factors were not adequately addressed in 3 studies (Hong 2005, 3 Toker, 2013; Mishra, 2020). Measurement of outcomes was consistently well reported across studies, with the use of validated eye-tracking devices and appropriate task settings. The overall risk of bias was low in most studies, although some studies (Toker, 2013; Mishra, 2020) lacked sufficient detail in minimizing potential biases. Two authors independently assessed the risk of bias (ALM and CB). Disagreements were resolved through a consensus after discussion with a third author. The methodological quality of the included studies was summarized using the CASP checklist, as shown in Table 2 .

**Table 2:** Critical Appraisal Skills Program tool to assess the quality of the observational studies in this systematic review

Study	Clear research question	Appropriate design	Recruitment strategy	Confounding factors	Measurement of exposure and outcomes	Minimizing bias	Adequate follow-up	Results clearly reported	Applicability to Practice????? Results Clearly Reported ??
Estrin et al., 2 2024	Yes	Yes	Yes	Unclear	Yes	Yes	Not available	Yes	Yes
Hong et al., 3 2005	Yes	Yes	Unclear	No	Yes	Unclear	Not available	Yes	Yes
Yoo et al., 4 2024	Yes	Yes	Yes	Yes	Yes	Yes	Not available	Yes	Yes
Kullmann, 2021	Yes	Yes	Yes	Yes	Yes	Yes	Not available	Yes	Yes
Fischer, 2016	Yes	Yes	N/A	N/A	Unclear	Unclear	Not available	Yes	Yes
Toker, 2013	Yes	Yes	No	No	Yes	Yes	Not available	Yes	Yes
Kelly et al., 15 1990	Yes	Yes	Yes	Yes	Yes	Yes	Not available	Yes	Yes
Mishra, 2020	Yes	Yes	Unclear	Unclear	Yes	Unclear	Not available	Yes	Yes

## DISCUSSION

### Clinical use of eye tracking in neurology

The present review supports the increasing integration of eye-tracking technology in neurology, especially in cognitive research. Eye movements, driven by neural commands from the frontal and supplementary eye fields to the superior colliculus, reflect essential cognitive processes such as attention, decision-making, and memory. 15 16 17 18 22 23 Understanding these eye behaviors provides insight into underlying cognitive deficits.

Although neurologists are now the main users of this technology, ophthalmologists were early adopters. They routinely identified abnormal eye movements (nystagmus, saccadic dysmetria, smooth pursuit deficits) as early indicators of neurological conditions like multiple sclerosis. 24 Early optical coherence tomography (OCT) findings linking fixation instability and foveation to MS prompted the use of eye-tracking to explore cognition-related abnormalities. 21 24

The value of saccadic tasks is especially clear. These rapid movements not only reflect how the brain shifts attention but also how visual information is encoded and remembered. 22 23 Saccade tasks like antisaccades and predictive saccades assess executive functions such as inhibitory control and visual prediction, both of which are commonly impaired in neurological conditions. 17

Eye-tracking also reveals differences in smooth pursuit, the ability to follow moving objects smoothly. Studies on schizophrenia patients demonstrate that their difficulties in this domain often reflect broader impairments in cognitive prediction and attention, in addition to core psychotic symptoms. 15 25 26 27

### Cognitive neurology implications

Neurodegenerative and cognitive disorders, including PD, may change eye movements patterns. 23 Parkinson's disease patients often present with hypometric prosaccade and short-latency saccades, indicating an issue in their inhibitory control. 28 Besides, they are able to accurately track moving objects but unable to move eyes toward stationary targets. 29 We believe that those deficits may interfere in daily activities such as watching television, reading and interacting with others. 30 This difficulty reflects broader cognitive impairments related to attention and visual processing. 5 20

Regarding Alzheimer's disease, patients may have difficulty with predictive saccades, which are essential for anticipating the movement of objects and maintaining attention. 20 Eye tracking is also capable of measuring visual memory by presenting a figure and subsequently assessing visual recall through tracking the participant's gaze patterns. We believe this could serve as a biomarker for memory recall. Memory and attention can be measured with an eye tracking device by showing a target stimulus (a red circle) on one side of the screen, while an animal animation with sound acts as a reward when the child gazes at the target. At the start of the trial, a gaze-contingent fixation stimulus appears in the center of the screen, allowing the child to adjust the speed of their saccades based on their attention level. Yoo et al. 4 also employed this technique to monitor attention in patients with ADHD.

Traumatic brain injury can also alter eye movements, particularly saccadic movements, increasing their latencies, with a delay in initiating it, affecting reaction times. 31 This may be explained by possible lesions to the corpus callosum and superior colliculus, important structures in saccade function, usually at risk of diffuse axonal injury in TBI. 32 Given that patients who suffer from TBI are often admitted to the intensive care unit (ICU), conducting studies in this acute setting can be challenging due to the complexity of their condition and the constraints of the hospital environment. We believe that utilizing portable eye-tracking devices would facilitate research in these critical care settings, enabling more accessible assessments of eye movements and cognitive function without the need for extensive infrastructure or equipment that may not be readily available in ICUs.

Another promising application of eye tracking, already in use and with the potential to facilitate cognitive diagnosis, is its application in patients with MS. 33 Cognitive impairment in MS can be challenging to detect, as it often manifests as subtle changes that are difficult to measure with traditional, extensive neuropsychological tests. 34 Multiple sclerosis frequently affects both motor and cognitive systems, including attention, processing speed, and executive function, all of which can be reflected in abnormal eye movements. 35 Multiple sclerosis-related demyelination can disrupt the neural pathways that coordinate visual processing and oculomotor control, resulting in measurable changes in eye movement patterns. Studies have shown that impaired performance in eye-tracking tasks, such as increased latencies or reduced accuracy in antisaccades, may indicate deficits in cognitive domains like executive functioning and attentional control. 36 Thus, eye tracking provides a valuable tool for early detection of cognitive decline in MS patients, allowing for more timely interventions and monitoring of disease progression. In this context, portable eye-tracking devices would be especially beneficial for studying the MS population, as they offer the possibility for more accessible, real-time assessments in clinical and everyday settings.

### Technological specifications and advances

Eye-tracking devices, whether portable or stationary, function through: i) infrared illumination (light reflects off the cornea and is captured by cameras and sensors); ii) pupil center corneal reflection (devices detect the position of the pupil and the corneal reflection to understand where the user is looking); iii) VOG: real time processing eye-tracking metrics (fixation points, saccades, and pupil dilation). This technology is widely used across industries like psychology, marketing, user experience design, and clinical neurology. 37 38 Those pieces of equipment analyze responses based on the frequency of acquisition, which indicates how many times per second the eye-tracking system captures data. For example, a sampling frequency of 60 Hz means the device takes 60 measurements of eye position per second. By sampling eye position at high frequencies, these devices can provide detailed insights into visual attention and cognitive processes. For instance, a higher frequency of acquisition allows researchers and clinicians to observe rapid saccadic movements and smooth pursuit dynamics in real-time, facilitating a better understanding of how individuals interact with visual stimuli. By employing devices with adequate sampling rates, practitioners can enhance diagnostic accuracy and tailor interventions to meet individual patient needs.

All devices process the images captured by the cameras using algorithms to identify key eye features of eye movements while this data are then translated into coordinates that represent where the user is looking on a screen. 39 Various metrics can be derived from the data, including gaze duration, blink rate, saccades and other patterns that provide insights into visual attention and cognitive processes. 40 All portable devices mentioned in the present study are connected devices, and they are linked to computers, tablets, or smartphones for real-time data analysis and display, which are more technological than previous standalone devices designed for research setting, with limited display options and low storage for data collection.

**Figure 2:**
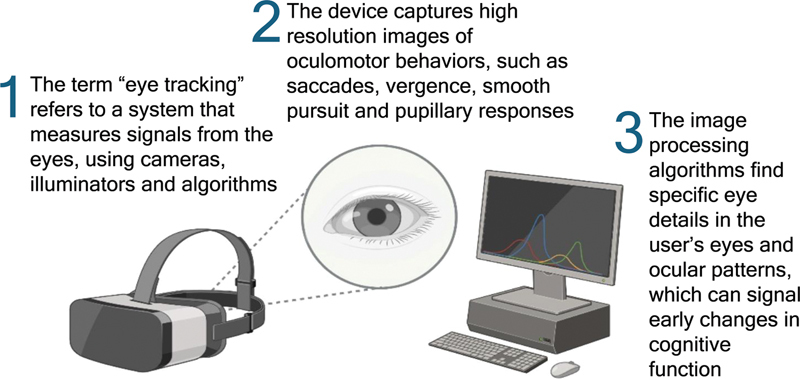
Exemplification of portable eye-tracking device.

The advancement of portable eye-tracking technology has rendered this domain significantly more approachable. Previous iterations of these systems were characterized by their considerable size, high expense, and limitations in functionality. Contemporary devices, which are both lightweight and ergonomically designed, possess the capability to accurately record high-frequency data in practical environments ( Figure 2 ). 13

**Figure 3:**
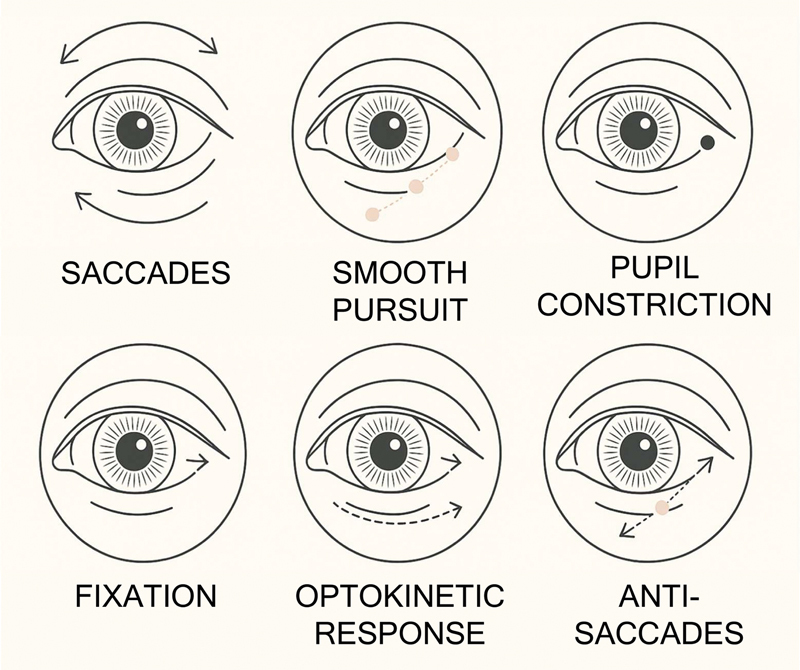
Illustration of oculomotor responses detected by eye-tracking technology: saccades, smooth pursuit, pupil constriction, fixation, optokinetic response, vergence, and antisaccades.

Devices operate using infrared illumination, pupil center-corneal reflection, and VOG, which detect where a person is looking and how the eyes move. 37 38 The integration of these technologies facilitates precise tracking of eye movements, allowing for the analysis of visual focus and cognitive functions ( Figure 3 ). By employing high-speed cameras to capture eye movements, researchers can gain valuable insights into mental processes, attention direction and visual memory in real time. High acquisition frequencies (e.g., 60 Hz or more) allow researchers to track even micro-movements in saccades and smooth pursuit in real time, making it easier to study cognition and visual attention accurately.

Modern portable devices process eye data through embedded algorithms that extract fixation points, blink rates, gaze duration, and saccade metrics. 39 40 They connect directly to tablets, phones, or computers, enabling real-time data visualization and eliminating the need for high-end laboratory equipment.

Devices like Neurolign DX 100, cited in 2 studies from our review, use high-speed video cameras to track eye movement precisely, capturing up to 100 to 250 frames per second. Similar devices are compact and easy to transport, with some devices weighing less than 600 g, allowing them to be used in various environments, including clinics, hospitals, and research facilities. Neurolign DX100 is more specialized for medical and neurological assessments, while Tobii, used in the autism study in this review, is more versatile. 2 Tobii offers greater flexibility for various applications outside of healthcare, measuring eye-tracking not only for medical purposes, but also for marketing research and commercial applications. SeeSo SDK is also an eye-tracking software, but developers need technical expertise to implement and customize the SDK within their applications. 4 It offers flexibility but may require more effort to set up and optimize.

## Limitations and future directions

Despite their promise, widespread adoption of eye-tracking in clinical practice faces obstacles. In countries like Brazil, financial constraints, lack of infrastructure, and the need for clinician training limit access. There is also a gap in interdisciplinary integration. Combining eye tracking with electroencephalogram, functional MRI, or other biomarkers could significantly enhance diagnostic precision, but few studies have explored this. 41

Normative eye-tracking databases, such as the one being developed by Kulmann et al., provide a foundation for population-wide screening. 13 Establishing baseline metrics for eye behavior will allow deviations to be used as clinical indicators of disease. Our review, which focused exclusively on portable systems, confirms their viability for these broader efforts.

As the technology matures, integrating eye tracking into routine neurological assessments could transform the early detection and tracking of cognitive decline. Potential applications include cognitive rehabilitation, ICU cognitive monitoring, and even remote assessments through smartphone-connected systems.

In conclusion, eye-tracking devices are promising in neurology for their ability to provide precise insights into eye movements, particularly affecting cognitive disorders. Eye tracking in MS patients is promising to better understand their cognitive patterns, such as processing speed, and other neurodegenerative diseases. Portable devices should be further explored and studied, as their accessibility facilitates research and makes it applicable in various contexts, including hospitals lacking extensive research infrastructure.
